# Pin1 Null Mice Exhibit Low Bone Mass and Attenuation of BMP Signaling

**DOI:** 10.1371/journal.pone.0063565

**Published:** 2013-05-10

**Authors:** Zhong-Jian Shen, Jie Hu, Aktar Ali, Johanne Pastor, Kazuhiro Shiizaki, Robert D. Blank, Makoto Kuro-o, James S. Malter

**Affiliations:** 1 Department of Pathology, University of Texas Southwestern Medical Center, Dallas, Texas, United States of America; 2 Department of Internal Medicine, University of Texas Southwestern Medical Center, Dallas, Texas, United States of America; 3 Department of Medicine, University of Wisconsin School of Medicine and Public Health, Madison, Wisconsin, United States of America; Wageningen University, The Netherlands

## Abstract

Bone is constantly formed and resorbed throughout life by coordinated actions of osteoblasts and osteoclasts. However, the molecular mechanisms involved in osteoblast function remain incompletely understood. Here we show, for the first time, that the peptidyl-prolyl isomerase PIN1 controls the osteogenic activity of osteoblasts. *Pin1* null mice exhibited an age-dependent decrease in bone mineral density and trabecular bone formation without alteration in cortical bone. Further analysis identified a defect in BMP signaling in *Pin1* null osteoblasts but normal osteoclast function. PIN1 interacted with SMAD5 and was required for the expression by primary osteoblasts of osteoblast specific transcription factors (CBFA1 and OSX), ECM (collagen I and OCN) and the formation of bone nodules. Our results thus uncover a novel aspect of the molecular underpinning of osteoblast function and identify a new therapeutic target for bone diseases.

## Introduction

From the late stages of embryonic development throughout adult life, bone is precisely remodeled by the coordinated actions of two opposing type of cells, osteoblasts and osteoclasts. Osteoblasts deposit calcified bone matrix, and osteoclasts resorb it. Imbalance in the differentiation and/or function of either cell type will deregulate remodeling, leading to bone diseases such as osteoporosis or Paget’s disease [1]. Many genetic studies have clarified the molecular cascades controlling osteoclast differentiation and function. However, the molecular control of osteoblast differentiation and function is not fully understood. Osteoblast commitment and differentiation are regulated by BMPs and WNT signaling that control the sequential actions of CBFA1/(RUNX2 or OSF2) and Osterix (OSX), two osteoblast-specific transcription factors [2]. Once expressed, these transcription factors drive osteoblasts to synthesize and secrete bone extracellular matrix (ECM) including type I collagen, osteocalcin and bone sialoprotein.

In addition to the cell-autonomous control by the transcription factors, osteoblasts are also regulated by many paracrine growth factors, most notably members of the TGF-β/bone morphogenetic protein (BMP) superfamily. The BMP family comprises at least 20 secreted cytokines and includes BMP2 that strongly induced ectopic bone formation after its expression in mouse muscle. BMPs can also initiate WNT signaling by inducing autocrine expression of WNT1/3a that in synergy with BMPs, promote osteoprogenitor proliferation and expansion [3]. BMP signaling also regulates cartilage, heart, kidney, gut, lung and brain development.

Signaling by BMPs is initiated by type I and II receptors and downstream SMAD1/5/8 (R-SMADs) that initially accumulate in the cytoplasm but translocate into the nucleus after activation [4]. The BMP-specific SMADs contain N-terminal MH1 and C-terminal MH2 domains, which are connected by a proline-rich linker. The R-SMADs are directly activated by type I receptor-mediated phosphorylation of the MH2 domain. This facilitates R-SMADs oligomerization and association with Co-Smad4 through the MH2 domain followed by translocation to the nucleus where the MH1 domain binds specific DNA sequences (GCCG/TGTGC) of target genes. Inhibitory SMAD6/7 blocks the phosphorylation of R-SMADs by interacting stably with the type I receptor and also mediates receptor degradation via the proteasome. Depending on cell type, BMP can also activate MAPKs such as JNK and p38. Signaling crosstalk likely integrates the input from diverse paracrine signals to allow for cooperative gene activation by R-SMADs and MAPK substrates c-JUN, JUN-B, and ATFs. For example, the cAMP-PKA, MAPK and PKC pathways have been shown to mediate BMP2-induced activation of CBFA1 and OSX, and to be affected by a variety of factors such as hormones, cytokines, and mechanical loading. However, it has been unclear how these factors modulate BMP specific SMAD actions.

Recently, the role of PIN1, a peptidyl-prolyl isomerase in regulating phosphorylation dependent signaling has received increased attention [5]. We and others have shown that PIN1 plays an important role in cytokine production and signaling in immune cells [6], [7], [8]. PIN1 isomerase activity was markedly increased by multiple cytokines as well as by kinase agonists such as PMA and hyaluronic acid, leading to functional changes in kinases, phosphatases, and downstream effectors involved in cell death decisions and mRNA abundance (e.g. GM-CSF, PTH, and IP-10). PIN1 was essential for KSRP mediated PTH mRNA decay such that mice lacking *Pin1* expressed elevated serum PTH levels, mimicking hyperparathyroidism. PIN1 also regulates TRF1 protein stability and the conformation of estrogen receptor alpha (ERα), the androgen receptor and the steroid receptor coactivator 3 required for receptor-mediated transcription [9], [10], [11]. Moreover, several key signaling molecules (e.g. SMAD2/3, NF-kB, WNT, ERα and AP-1) and cytokines (e.g. IL-1, TNF, IL-4, GM-CSF, TGF-β, FGF, and PDGF) that are implicated in bone formation, interact with and are regulated by PIN1. These data suggest that PIN1 might play an important role in bone metabolism. In this study, we present new evidence that PIN1 associates with SMAD5 and is required for the expression of osteoblast specific transcription factors and the formation of bone nodules in vitro. PIN1 null mice exhibited significantly reduced BMD and trabecular bone volume. Our findings indicate that PIN1 is crucial for the bone remodeling and suggest a new molecular target for the treatment of bone diseases.

## Materials and Methods

### Materials

Recombinant human BMP2 was purchased from R&D. Ascorbic acid, Alizarin Red S, Vitamin D3, Dexamethason, p-Nitrophenyl phosphate, Fast Red Violet LB salt, Sodium Tartrate, Naphtol AS-MX phosphate Disodium Salt were from Sigma. Mouse NTX ELISA Kit was from CUSABIO. Mouse TRAP-5B Kit was from MyBioSource. Vt.D3 Kit was from Immunodiagnostic Systems (IDS). Protease Inhibitor Cocktail Set III was from Calbiochem. Monoclonal anti-β-actin (Ab-1) was from Oncogene Research Products. Horseradish peroxidase–conjugated anti-rabbit (secondary antibody; NA934V) and the enhanced chemilumiscence ECL immunoblot detection system were from Amersham-Pharmacia. Anti-SMAD 1 and 5 were from Abcam. Anti-PIN1 was from Santa Cruz. SYBR Green PCR Master Mix was from Applied Biosystems. PCR primers (Table S2) were designed with Primer Express software and purchased from IDT, Inc. Mouse MC3T3E1 and human MG-63 osteoblast cell lines were purchased from ATCC.

### *Pin1* ^−/−^ mice

*Pin1* ^+/−^ mice on a pure C57BL/6J background were obtained from T. Means (Duke University, Durham, North Carolina, USA) [7]. The mice were generated by deleting all 4 exons of *Pin1*. Full knockout (KO) mice were produced by crossing heterozygotes. Animal care was carried out in strict accordance with the recommendations in the Guide for the Care and Use of Laboratory Animals of the National Institutes of Health. Our protocol was approved by the Committee on the Ethics of Animal Experiments of the University of Texas Southwestern Medical Center (Permit Number: 2011-0139). All surgery was performed under sodium pentobarbital anesthesia, and all efforts were made to minimize suffering.

### Reverse Transcription and Real-time PCR

RNA was extracted with TriReagent. cDNA Quantitative PCR was performed with a SYBR PCR master mix with the primers shown (Table S2). An ABI 7500 thermocycler (Applied Biosystems, Foster City, CA) was used for 45 cycles of PCR. ΔCT calculates the differences between target CT values and the normalizer (housekeeping gene) for each sample: ΔCT =  CT (target)−CT (normalizer). The comparative ΔΔCT calculates the differences between each sample ΔCT value and the baseline ΔCT. The comparative expression level (fold changes) was obtained transforming the logarithmic values to absolute values using 2^−ΔΔCT^. All data from untreated control cells was normalized to 100%.

### ALP Activity Assay and Serum Biochemistry

Osteoblasts differentiated from bone marrow were rinsed twice with ice-cold phosphate-buffered saline and scraped into 10 mM Tris-HCl containing 2 mM MgCl_2_ and 0.05% Triton X-100, pH 8.2. The cell lysates were briefly sonicated on ice after two cycles of freezing and thawing. Aliquots of supernatants were subjected to ALP activity measurement [12] and protein assay according to Bradford’s method. In brief, the lysate was mixed with assay buffer containing 10 mM ***p***-nitrophenyl phosphate in 0.1M sodium carbonate buffer, pH 10, and 1 mM MgCl_2_, followed by incubation at 37°C for 30 min. After adding 1 M NaOH, the amounts of ***p***-nitrophenol liberated were measured by a spectrophotometer. Total serum ALP activity (international units), calcium and phosphate concentrations were measured on an Ortho Clinical Vitros 250 Chemistry System in the Mouse Metabolic Phenotyping Core at UT-Southwestern Medical Center, and Vt.D3 by ELISA kit.

### Measurement of Bone Mineral Density

Bone mineral density (BMD) (g/cm^2^) was measured based on dual-energy X-ray absorptiometry (DXA) using an apparatus specifically designed for small animals (PIXI: GE Lunar) equipped with a computer. For whole body BMD, mice were anesthetized by i.p. injection of pentobarbital and immediately subjected to BMD measurement. Calibration was performed using a standard block according to the manufacturer’s instruction. After euthanasia, blood was drawn for serum biochemistry and isolated femurs were subjected to ex vivo BMD measurement. Femurs were also formalin-fixed and after decalcification, representative sections prepared and stained with H&E, trichrome and anti-PIN1.

### uCT

High resolution microcomputed tomography was used to evaluate bone dimension, shape, volume and microarchitecture in the entire femur and lumbar vertibrae (L1-L6). Bone was scanned with a Siemens MicroCAT-2 (Department of Radiology, Madison, WI) at 35 KeV with a slice increment of 9 µm. CT images were reconstructed with an isotropic voxel size of 9 µm, and the gray-scale images were segmented using a constrained three-dimensional (3D) Gaussian filter (σ = 0.8, support = 1.0) to remove noise, and a fixed threshold (35% of maximal gray scale value) was used to extract the structure of mineralized tissue. Constructed 3D images were sliced with Amira software for 2D BV/TV analysis and anatomical location for each bone was determined by skeletal landmark. Three slices (27 µm apart)/bone were imaged and used for quantification by ImageJ software. An area of 0.54 mm^2^ (600 µm×900 µm) in the distal metaphyseal region of the femurs or the fourth lumbar vertebrate (700 µm×700 µm) was chosen for the analysis [13].

### Bone Marrow Culture for Osteoclast Differentiation and TRAP Staining

Bone marrow cells from femurs were cultured in the presence of 1, 25 (OH)_2_ vitamin D3 (10 nM) and dexamethasone (100 nM) for 5 days. Cells were harvested and total RNA extracted for qPCR. For TRAP staining, cells were fixed with 10% formalin for 5 min then re-fixed with ethanol: acetone (50∶50 v/v) for 1 min and incubated in acetate buffer (pH 4.8) containing naphthol AS-MX phosphate (Sigma Chemical Co., St. Louis, MO), fast red violet LB salt (Sigma), and 50 mM sodium tartrate for 30 min at room temperature. TRAP positive multinucleated osteoclasts (greater than 3 nuclei) were counted and expressed as number/mm^2^.

### Immunoprecipitation and Immunoblots

Bone marrow derived osteoblasts, osteoblast cell lines or primary calvarial osteoblasts were lysed in NP-40 buffer (1% Triton-X 100, 0.5% NP-40, 150 mM NaCl, 10 mM Tris-pH7.4, 1 mM EDTA and 1 mM EGTA). For immunoprecipitation, 2–5 µg antibody was added to each sample, followed by incubation for 2–4 h at 4°C. Protein G–agarose beads (Sigma-Aldrich) were added and incubation was continued overnight. Pellets were washed five times with lysis buffer and beads were dissolved in SDS-PAGE loading buffer for immunoblot analysis. The proteins were transferred onto nitrocellulose membranes and, probed with primary and secondary antibodies. Protein bands were detected using enhanced chemiluminescence (ECL).

### Primary Calvarial Osteoblast Culture

Mouse calvarial osteoblast cultures from young mice (2–3 week-old) were generated as described [14] and induced to undergo osteogenic differentiation in the presence of 10 mM β-glycerophosphate and 50 µg/ml ascorbic acid for 20 days before use.

### PIN1 Activity Assay

Protease-coupled PIN1 activity was measured with 1 µg cytoplasmic protein [6] after incubation in buffer with a glutamine-proline–containing pentapeptide target modified with a carboxy-terminal nitroanaline. After the PIN1 mediated conversion from *cis* to *trans*, the nitroanaline was cleaved by chymotrypsin and the resulting absorbance was measured at 390 nm (*A*_390_). Dominant negative TAT-WW peptides (WW domain of PIN1 fused to a cell penetrating TAT peptide) [6], [7], [8] were used to inhibit PIN1 activity or to disrupt endogenous PIN1 interaction with its targets. TAT-GFP served as control for cell penetration.

### Osteoblast Differentiation and Mineralized Nodule Formation Assay

Bone marrow cells from 2–3 week-old mice were cultured for 21 days in α-minimal essential medium (10% fetal bovine serum, 10 mM β-glycerol phosphate, and 50 µg/ml ascorbic acid) (changed every 3 days) [13]. After removal of the non-adherent cell population, adherent cells were trypsinized and counted to calculate differentiated osteoblast numbers. For Alizarin Red staining, part of the adherent cell population were rinsed in Ca^2+^/Mg^2+^ free phosphate-buffered saline and fixed for 5 min in 10% formalin/saline. The cells were then incubated with Alizarin Red (0.1% in saline) solution for 3–7 min followed by several rinses with water before scoring the bone nodules.

### Statistical Analyses

Statistical evaluations of the data were conducted by using Student’s t test for per-comparison analysis. P<0.05 is considered statistically significant. The data were presented as mean ± S.D.

## Results

### PIN1 is Expressed in Bone

**Figure 1:**
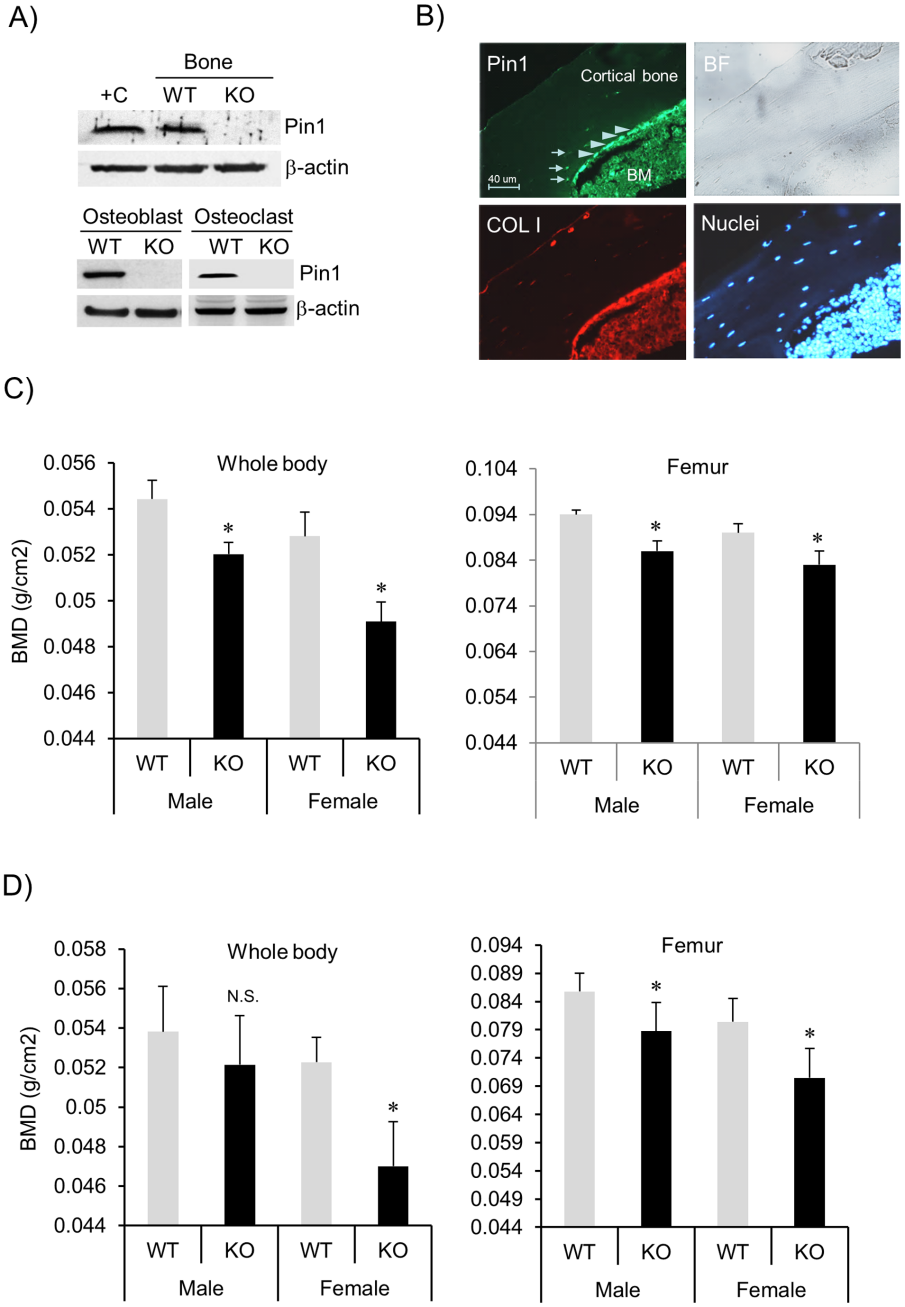
Bone mineral density (BMD) is decreased in *Pin1* KO mice. (**A**) PIN1 is expressed in mouse bone, osteoclasts and osteoblasts. Top: Immunoblot of total cell lysates from human eosinophils (+C) and humerus bone of PIN1 WT and KO mice. Bottom: Immunoblot of bone marrow derived osteoblasts and osteoclasts. (**B**) Femoral bone was triple-stained with anti-PIN1 (green), anti-COL-I (red) and Hoechst dye (blue) for visualization of nuclei. Bone from KO mice was stained negative for green (data not included). BM: bone marrow. Arrow head: Osteoblasts. Arrow: Osteocytes. BF: bright field. (**C**) Whole body (left) and femoral (right) bone mineral density (BMD) of 4 month-old wild-type and *Pin1* KO mice (littermate) were measured non-destructively by DXA system. For whole body BMD, we excluded skull and tail for each animal based on skeletal landmarks from the grey-scale images. Femoral BMD was measured ex vivo after isolating femurs from same mice. (**D**) BMD of 18 month-old mice was measured as in (C). *denotes p<0.05 by paired student t-test with 8 mice per group.

PIN1 is present in all tissues with highest levels found in brain, liver, muscle, respiratory tract, and germ cells. Its expression is often higher in proliferating cell populations [5], including many cancers from prostate, lung, ovary, cervix, brain, and skin [5]. Previously, we showed that PIN1 is highly expressed in immune and mesenchymal cells [6], [7], [8]. In order to determine the level of PIN1 in bone, homogenates from humerus (less bone marrow) of 2–3 week-old mice were immunoblotted. As seen (Fig. 1A, top), PIN1 was expressed in bone at a level comparable to that seen in freshly purified human eosinophils (+C, positive control). Next we determined if PIN1 is expressed by osteoblasts and/or osteoclasts. Cells were differentiated from bone marrow, lysed and subjected to western blotting. As shown (Fig. 1A, bottom), PIN1 is expressed in primary osteoblasts and osteoclasts. IHC confirmed PIN1 expression by osteoblasts in bone and bone marrow cells (Fig. 1B) but less so in osteocytes. Notably, the PIN1 expression in the human osteoblastic cell line MG-63 was much higher than normal bone (data not shown). These data indicate that PIN1 may play a role in osteoblasts, osteoclasts and bone metabolism.

### Bone Mineral Density is Decreased by *Pin1* Deficiency

Previously, PIN1 was shown to regulate PTH production and ERα function [9], [10]. Moreover, several key signaling molecules (e.g. SMAD2/3, NF-kB, WNT, and AP-1) that are implicated in bone formation, interact with and are regulated by PIN1 [15], [16]. To establish a role for PIN1 in bone, we performed radiography to determine whole body bone mineral density (BMD) in 4 month-old wild-type (WT) and *Pin1* KO mice (littermates). KO mice had no significant changes in body weight (p = 0.6124) (data not shown). Mice were anesthetized and immediately scanned with a PIXImus small animal dual-energy X-ray absorptiometry (DXA) system. Similar to published data, the BMD in females was slightly lower than male mice (0.0523-0.0542 g/cm2) (Fig. 1C, left) [17]. Mice lacking PIN1 developed mineralized bones but had a significant decrease (6–8%) in BMD (p<0.05) compared to wild-type littermates of both genders. We next measured BMD of isolated left and right femurs of the same mice to confirm the *in vivo* results. Similar to previously published data, femoral BMDs were much higher when measured *ex vivo* from WT or KO mice (Fig. 1C, left and right) [17]. However, femoral BMD was still lower (p<0.05) in KO compared to WT littermates (Fig. 1C, right). We repeated this experiment using 18 month-old animals. Interestingly, PIN1 KO mice displayed greater age-dependent whole body BMD loss (1.9% vs 1.6% in male, 9.4% vs 5.1% in female) (Fig. 1D, left) and femoral loss (12.5% vs 9.5% in male, 17.8% vs 10.1% in female) than WT (Fig. 1D, right). Of note, the age-dependent BMD loss by PIN1 deficiency was statistically significant in femoral bone and more accelerated in females. These results imply that PIN1 is required to maintain normal BMD and possibly for sex hormone dependent signaling as well in bone.

### Trabecular Bone Volume is Decreased by *Pin1* Deficiency

**Figure 2:**
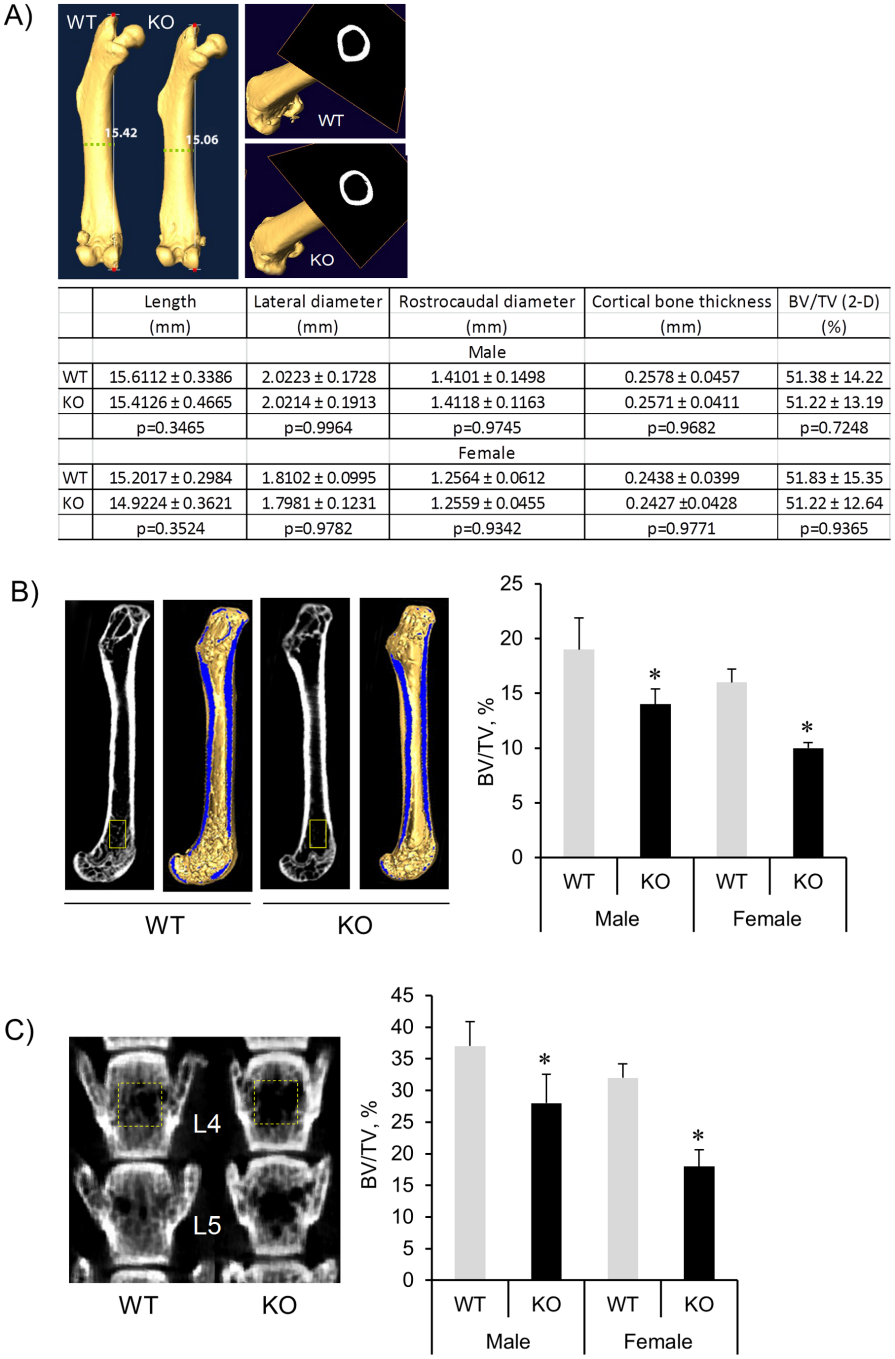
Trabecular bone volume is reduced in *Pin1* KO mice. 3D µCT reconstruction and slice of representative femurs and vertebral sections from 4 month-old mice. (**A**) Femoral bone parameters were determined with 3D construction using Amira and ImageJ softwares. Upper right: Cross-sectional slice at mid-diaphysis from each femur. (**B** and **C**) Trabecular bone volume (BV/TV) of the distal end of femurs (**B**) and the lumbar vertebra (L4) (**C**) was quantified as described in the Methods after slicing the 3D construction in longitude direction. *denotes p<0.05 by paired student t-test with 8 mice per group.

BMD is a reflection of both cortical and cancellous bone mass *in vivo*, and is the single best predictor of the risk of osteoporotic fractures. Therefore, we analyzed the cortical and trabecular bone compartments in isolated femurs after scanning with peripheral quantitative µCT. The acquired data set were reconstructed to 3D images using Amira software. After re-slicing the bone image in lateral and longitude directions, the femoral bone length, inner and outer circumference in mid-diaphysis, cortical thickness and volumes and trabecular bone volumes in the distal metaphysis were quantified. This analysis revealed no significant differences in those parameters (Fig. 2A) except the trabecular bone volumes (BV/TV) in distal femurs was significantly decreased in KO compared to age- and sex-matched WT littermates (Fig. 2B). µCT morphometric analysis of distal femurs identified a significantly reduced trabecular bone thickness and trabecular number without marked changes in trabecular spacing (Fig. S1). Similar results were obtained in trabecular-rich, fourth and fifth lumbar vertebra (L4 and L5) (Fig. 2C and data not shown). Interestingly, there was a high positive correlation (r = 0.8286−0.8910, p<0.001) between whole femoral BMD (Fig. 1C, right) and trabecular BV/TV (Fig. 2B, right) in both genotypes particularly in female KO mice (r = 0.8910). These data suggest the lower BMD seen in KO mice was likely due to the loss of trabecular (cancellous) bone throughout the skeleton.

### Serum Ca2+, Pi and Vt.D3 Concentration and ALP Activity are Altered by *Pin1* Deficiency

**Figure 3:**
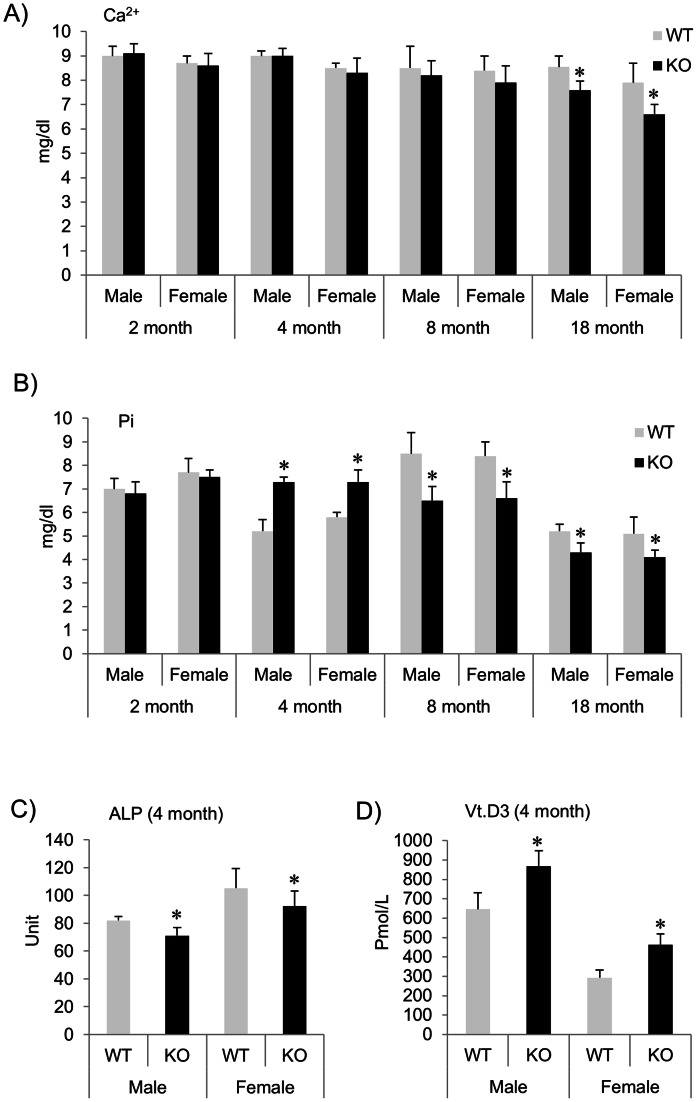
Ca^2+^, Vt.D3 and Pi concentrations and ALP activity are abnormal in the sera of *Pin1* KO mice. Ca^2+^ (**A**) and Pi (**B**) concentrations (mg/dl) were measured in serum from *Pin1* WT and KO mice (2, 4, 8 and 18 month-old), and ALP activity (**C**) and Vt.D3 (**D**) from 4 month-old mice. The serum ALP activity is expressed in international units (IU). *denotes p<0.05 by paired student t-test with 5 mice per group.

Bone formation and mineralization are greatly influenced by the serum Ca^2+^ and Pi which form hydroxyapatite crystals in bone. Bone stores and releases them in response to physiological demand and their levels are under tight hormonal control on target organs such as kidney, bone and intestine. Serum ALP is of most interest in the differential diagnosis of hepatobiliary versus bone disorders associated with increased osteoblastic activity. Given those attenuated BMD values and trabecular BV/TV, we measured the Ca^2+^, Pi and Vt.D3 concentrations and total ALP activity in serum. The serum Ca^2+^ and Pi levels were similar in 2 month-old WT and KO littermates but lower (15–17%) in 8-month and 18-month old KO mice compared to WT (Fig. 3A and 3B). Interestingly, in comparison to younger mice (2 month-old) the levels of Pi were markedly increased (20–25%) in 4 month-old KO compared to WT mice (Fig. 3B). Regardless of this change, in KO mice, the activity of ALP was significantly decreased whereas the level of active Vt.D3 was increased (by 30–40%) compared to WT littermates in both genders (Fig. 3C and 3D). These data suggest that PIN1 is required for systemic Ca2^+^ and Pi homeostasis involving multiple hormonal pathways. These changes may also partly account for the loss of trabecular bone in vivo or result from a functional disorder of osteoblastic bone formation or/and osteoclastic bone resorption by PIN1 deficiency.

### *Pin1* Deficiency does not Affect Osteoclast Differentiation and Function

**Figure 4:**
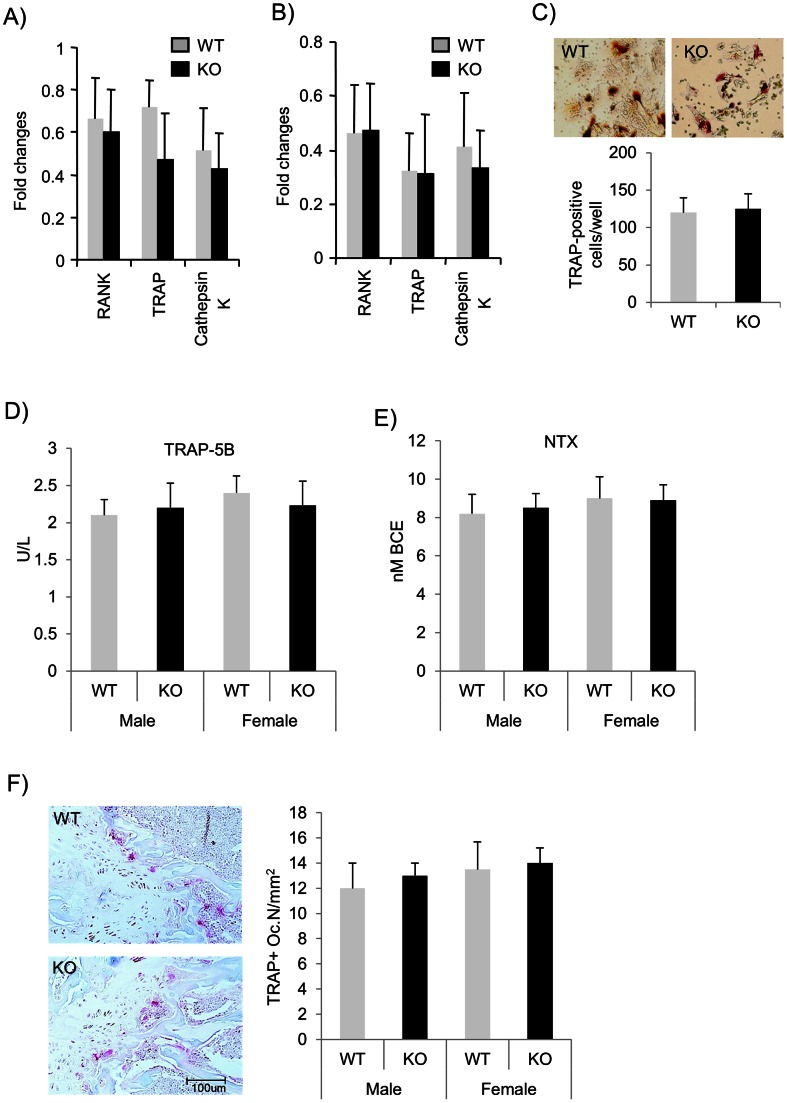
PIN1 deficiency does not affect osteoclast generation and function. (**A**) qPCR for RANK, TRAP and cathepsin K mRNA in humeri. (**B**) Bone marrow cells were cultured in the presence of osteoclast differentiation medium for 5 days prior to qPCR as in (A). (**C**) Osteoclasts (>3 nuclei) were stained for TRAP after 5 days of culture (12-well plate) and positive cells counted. (**D** and **E**) Osteoclast markers TRAP-5B and NTX were measured in sera. (**F**) TRAP staining (red) of osteoclasts in femoral bone. Error bars indicate mean ± SD of 8 animals per group.

**Figure 5:**
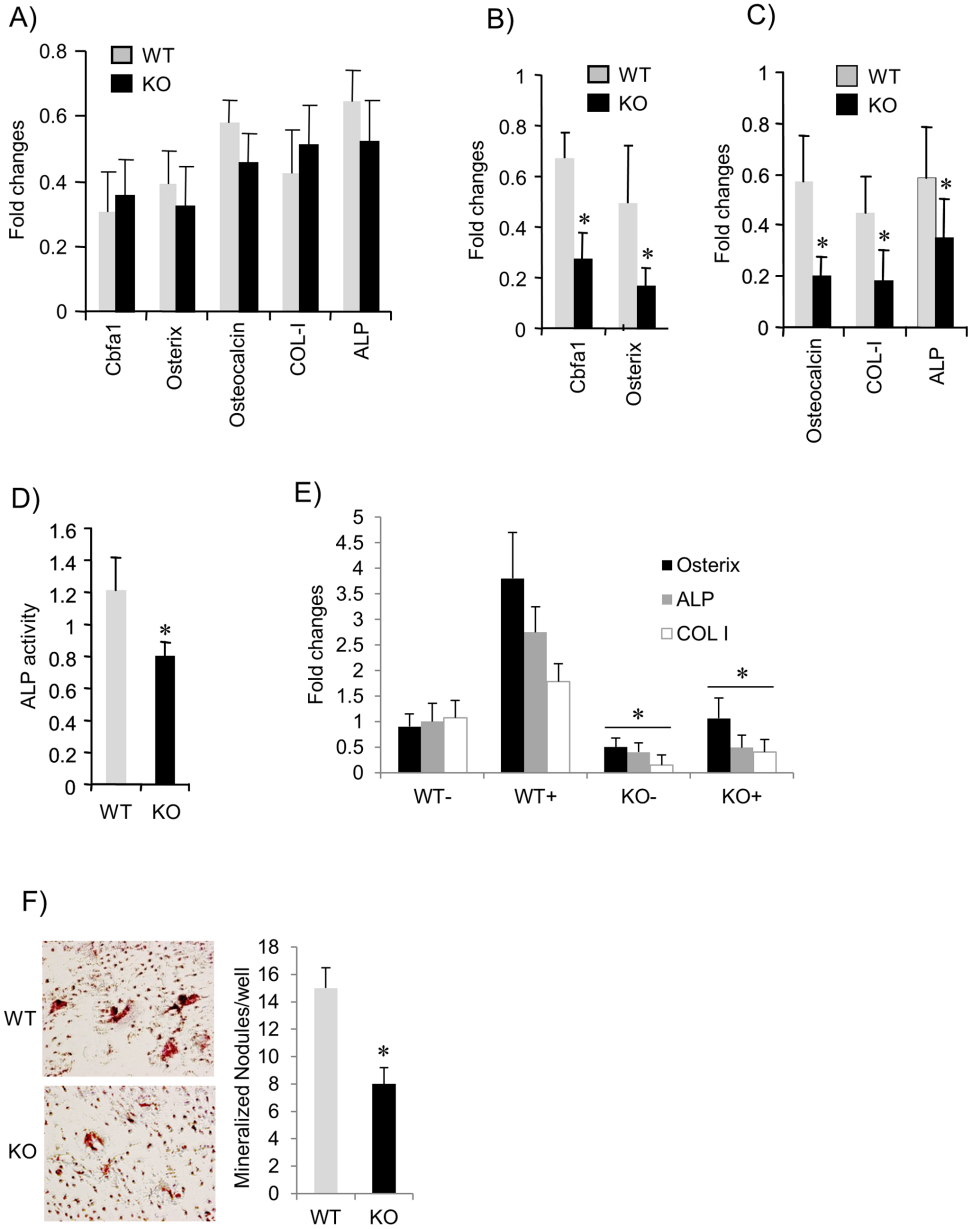
Osteoblast function and bone formation are impaired by *Pin1* deficiency. (**A**) RNA was extracted from humeri and subjected for qPCR for the genes indicated. (**B, C**) Expression of osteoblast-related genes by bone marrow-derived osteoblasts. Bone marrow cells were cultured in the presence of BMP2 for 10 days prior to qPCR for the expression of transcription factors (CBFA1 and OSX) and ECM (type I collagen, osteocalcin and ALP). (**D**) ALP activity in bone marrow-derived osteoblasts was measured (see Methods) and normalized to total protein. (**E**) Primary calvarial osteoblasts were cultured in the presence (+) and absence (−) of BMP2 for 3 days. Total RNA was subjected to qPCR for the genes indicated. (**F**) Bone marrow cells from *Pin1* WT and KO animals were cultured in the presence of mineralization medium (vitamin C and β-glycerol phosphate) for 21 days. Mineralized nodules were stained with Alizarin red S and the number of calcified nodules counted by light microscopy. For panel (**E**), KO- group has been compared to WT− and KO+ to the WT+ groups. The data represent one of 5 independent experiments. Error bars indicate mean ± SD of 5 animals per group. *denotes p<0.05 by paired student t-test.

The lower BMD and trabecular bone mass in *Pin1* KO mice was possibly due to a decrease in the numbers and/or function of osteoblasts or increased activity of osteoclasts. We first assessed the expression of the key osteoclast marker genes RANK, TRAP and cathepsin K. RANK is a member of the TNFR family that acts as a receptor for RANKL on osteoblasts and dendritic cells. Mutation in RANK cause defects in osteoclastogenesis, eventually causing osteopetrosis. qPCR analysis (Table S2) of whole humerus indicated no difference in the basal level of these marker genes between WT and KO mice (Fig. 4A). Next we addressed whether *Pin1* deficiency influences osteoclastogenesis and function. Bone marrow cells from femurs were cultured in the presence of osteoclast differentiation medium for 5 days. Gene expression was examined by qPCR and TRAP positive multinucleated osteoclasts (greater than 3 nuclei) were counted. This analysis also revealed no major differences between WT and KO osteoclasts either in the cell density or absolute numbers (Fig. 4B, Fig. 4C, and data not shown). Finally, neither the expression of serum osteoclast markers (TRAP-5B for osteoclast numbers and NTX for osteoclast function) nor the numbers of TRAP positive cells in femoral bone differed between genotypes (Fig. 5D–F), suggesting PIN1 does not affect osteoclast differentiation and function.

### *Pin1* Deficiency Attenuates Osteoblast Differentiation and Function

To date, the function of PIN1 in osteoblasts and bone formation has yet to be characterized. To delineate possible mechanisms underlying those phenotypes shown above, we asked whether PIN1 deficiency influences the maintenance of osteoblast-associated genes. Total RNA from humerus from strain-matched, WT and KO littermates (2–3 week-old) was subjected to real-time PCR analysis. We found that CBFA1, OSX, type I collagen, ALP and OCN mRNA were present and similarly expressed (<2 fold difference) between genotypes (Fig. 5A). CBFA1 (earliest differentiation marker) and downstream OSX are osteoblast-specific transcription factors essential for the development of mature osteoblasts, and are thought to activate ECM genes such as type I collagen, OCN, and ALP for matrix mineralization [2]. Our data shown here indicate that the lack of PIN1 does not affect the expression of osteoblast specific genes in the bone of young mice.

Next we asked whether osteoblast differentiation was impaired by *Pin1* deficiency. Bone marrow stromal cells are the major source of osteoblasts for bone remodeling and repair in postnatal animals. BMP2 strongly induces osteoblast differentiation from bone marrow and stimulates osteoblast function, making this a widely used system to study target genes associated with osteogenesis. We cultured bone marrow cells in the presence of BMP2 (100 ng/ml) for 10 days and the expression of CBFA1 and OSX were determined by qPCR. Strikingly, CBFA1 and OSX mRNAs were significantly reduced (50–60%) in KO cells compared to WT (Fig. 5B). We also measured the levels of downstream differentiation markers such as ALP (early marker), type I collagen (early marker), and OCN (late marker and osteoblast-specific). As expected, all these genes were significantly decreased in KO cells to a similar extent as seen for CBFA1 and OSX (Fig. 5C). As ALP activity is critical for matrix mineralization in developing and remodeling bone, we measured its enzymatic activity using p-nitrophenyl phosphate as a substrate. As shown (Fig. 5D), the enzymatic activity was also significantly decreased (by 30%) in the absence of PIN1. We obtained similar results (data not shown) with bone marrow cultured in a medium containing ascorbic acid plus β-glycerophosphate, the chemical enhancers of mineralization that stimulate autonomous osteoblast differentiation and function. In order to understand the role of PIN1 in osteoblast differentiation or function and its response to BMPs, we isolated primary calvarial osteoblasts. The cells were cultured in differentiation medium before treatment with BMP2. We found that *Pin1* KO cells grow more slowly than WT (data not shown) in differentiation medium, and the baseline and BMP2 induced expression of characteristic, osteoblast phenotypic genes was significantly decreased (Fig. 5E). Thus, we conclude that PIN1 is essential for osteoblastic cell differentiation and function in bone.

### *Pin1* Deficiency Reduces Bone Nodule Formation *in vitro*

Mineralized bone nodules can be used as an *in vitro* surrogate to assess the differentiation and functionality of bone marrow-derived osteoblasts. To address whether PIN1 contributes to osteoblast function in bone formation, we cultured bone marrow cells for 21 days in the presence of osteogenic medium, prior to fixation and staining with Alizarin red S to visualize bone nodules. Consistent with previous reports [12], we observed numerous bone nodules in cultures from WT mice, that were much delayed and reduced (up to 50%) in number in KO cultures (Fig. 5F) despite only a modest reduction (10–15%) of differentiated cells (data not shown), indicating PIN1 is required for osteoblastic bone formation.

### PIN1 Interacts with SMAD5 in Osteoblasts

**Figure 6:**
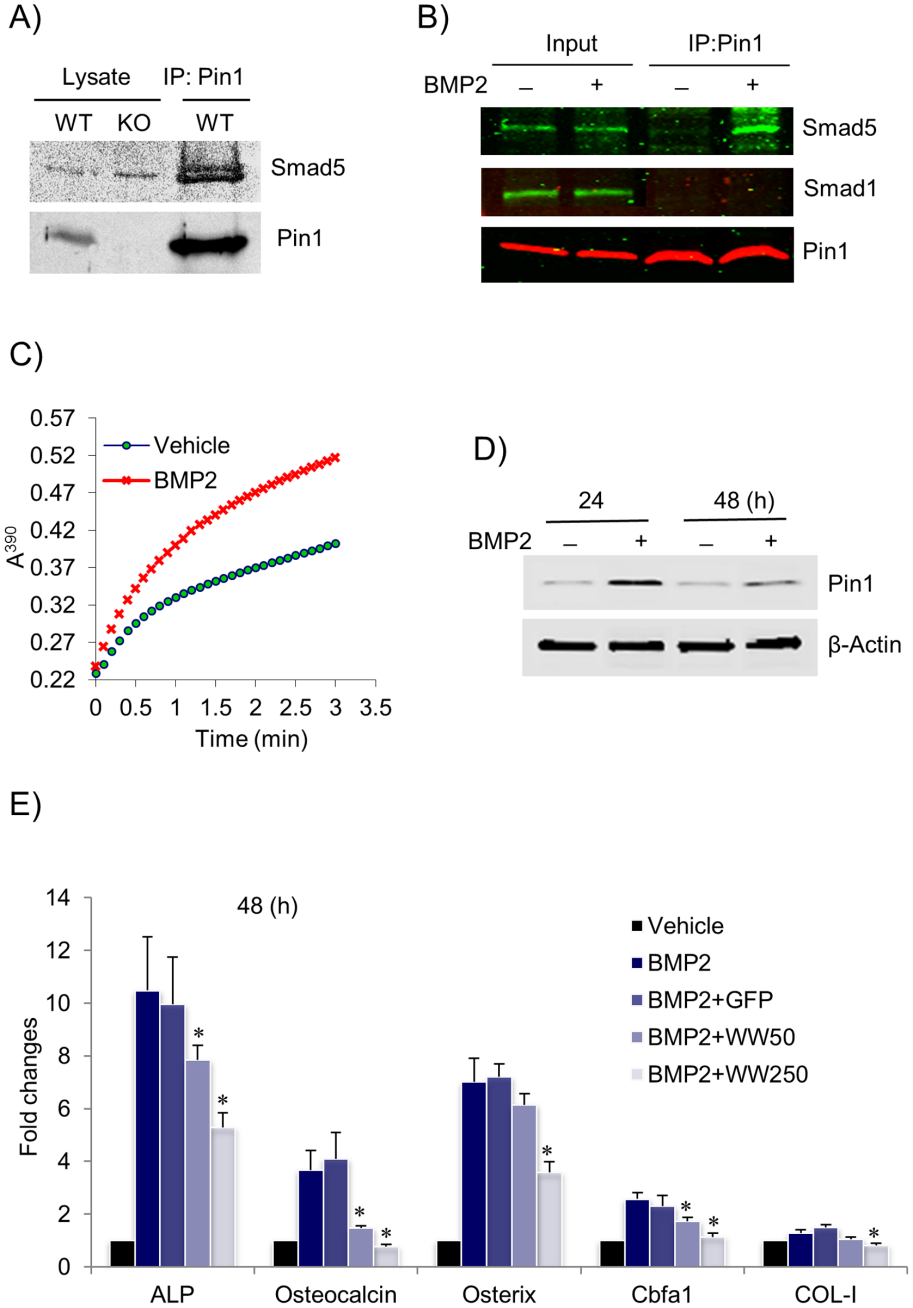
PIN1 interacts with SMAD5 and is regulated by BMP signaling. (**A, B**) PIN1 was co-immunoprecipitated with SMAD5 from osteoblast lysates**.** (**A**) Bone marrow cells were treated with BMP2 (200 ng/ml) for 10 days before immunoprecipitation with anti-PIN1 and immunoblot with the antibodies shown. (**B**) Primary calvarial osteoblasts were differentiated and then treated with BMP2 for 60 min before immunoprecipitation and immunoblot as in (A). (**C**) MC3T3E1 cells were treated with or without BMP2 for 10 min. Aliquot of lysate (1 µg protein) was subjected to protease coupled PIN1 isomerase assay. (**D**) MC3T3E1 cells were treated with or without BMP2 for 24 and 48 h before immunoblotting whole cell lysates with anti-PIN1. (**E**) MC3T3E1 cells were treated with BMP2 alone or together with dominant-negative TAT-WW (WW: 50 nM and 250 nM) peptide or control TAT-GFP (250 nM) for 48 h prior to qPCR for the genes shown. The data represent one of 3 independent experiments. Error bars indicate mean ± SD of 5 animals per group. *denotes p<0.05 by paired student t-test.

Our data strongly suggest that PIN1 might regulate BMP signaling in osteoblasts. SMAD1/5/8 are phosphorylated and activated by BMP2 and required for inducing differentiation of osteoprogenitor cells into osteoblasts as well as maintaining mature osteoblast function. Phospho-site database (http://www.phosphosite.org/) analysis revealed multiple conserved PIN1 recognition sites in SMAD1 and SMAD5 but none in SMAD8. Therefore, we performed co-immunoprecipitation and western blotting to determine if PIN1 physically interacts with SMAD1 and/or 5 in BMP2-differentiated bone marrow osteoblasts. We found that SMAD1 and 5 were equally expressed in WT and KO cells. Strikingly, PIN1 was reproducibly co-immunoprecipitated with SMAD5 (Fig. 6A) but not SMAD1 (data not shown). Next, we asked whether such interaction depends on ligand stimulation. We employed differentiated, primary calvarial osteoblasts that were cultured in the presence or absence of BMP2 for 60 min before analysis. As shown (Fig. 6B), PIN1 minimally interacted with SMAD5 in untreated cells that was dramatically increased after BMP2 treatment, indicating PIN1 is a partner for SMAD5 during osteoblastic cell differentiation and in mature osteoblasts. This data also suggests that BMP signaling may regulate PIN1 activity, which may in turn modulate Smad5 phosphorylation, nuclear translocation and DNA binding.

### PIN1 Isomerase Activity is Increased by BMP2

The regulation of osteoblast differentiation and bone formation is affected by many systemic (e.g. PTH, Vt.D3, leptin) and local factors (BMPs, IGF, WNT, VEGF, TGF). Recently, we found that cytokine treatment (e.g. IL-5, GM-CSF, IL-4, TGF-β) rapidly increases PIN1 isomerase activity in eosinophils as well as in T cells and fibroblasts [6], [8]. These data suggest that PIN1 PPIase activity may alter downstream signaling pathways through the conformational change of target molecules. We speculated that pro-/anti-osteogenic factors may also regulate PIN1 activity. To test this possibility we cultured human osteoblastic MG-63 cells that were treated with vehicle (diluent) or BMP2 (200 ng/ml) for 10 min prior to PIN1 activity assay. As shown in Fig. 6C, the PIN1 isomerase activity, but not the protein level (data not shown), was markedly increased by BMP2. Prolonged ligand exposure (24–48 h) (Fig. 6D) induced PIN1 expression as well as its activity (data not shown), indicating PIN1 itself is activated and controlled by BMP-receptor signaling, which might in turn regulate SMAD5 action.

### PIN1 Interaction with SMAD5 is Required for Osteoblast Function

In order to address the functional significance of the protein interactions, we employed dominant negative TAT-WW peptides (WW domain of PIN1 fused to a cell penetrating TAT peptide) [6], [7], [8] to disrupt endogenous PIN1 interaction with SMAD5. MC3T3-E1 osteoblasts were treated with BMP2 alone or together with TAT-WW or control TAT-GFP (50–250 nM) for 48 h prior to qPCR analysis. Untreated mouse MC3T3E1 cells expressed considerable amount of type I collagen but very low level of ALP (230,000 times less than collagen) (Table S1). Consistent with previously published data [12], BMP2 dramatically induced all marker genes (Fig. 6E) including ALP, that was suppressed by TAT-WW peptides in a dose dependent manner, suggesting the direct interaction between PIN1 and SMAD5 may play a crucial role for the expression of the osteoblast phenotype.

## Discussion

Accumulating studies have revealed that the prolyl isomerase PIN1 regulates diverse cellular processes including growth factor signaling, cell-cycle progression, cellular stress responses and neuronal function [5]. Accordingly, PIN1 has been implicated in the etiology of a large number of pathologic processes including cancer and Alzheimer’s disease. In this study, we demonstrate for the first time that PIN1 also control bone homeostasis suggesting it may contribute to the pathogenesis of skeletal diseases. We show that: 1) PIN1 interacts with BMP-specific SMAD5 and facilitates SMAD-dependent transcription of CBFA1 and OSX, the key transcription factors which determine osteoblast commitment, differentiation and function; 2) PIN1 is required for trabecular bone formation and maintenance of BMD and physiologic serum Ca^2+^/Pi and Vt.D3 levels in vivo; 3) PIN1 isomerase activity is increased upon BMP2 stimulation; and 4) PIN1 is dispensable for osteoclast generation and function. These data may ultimately yield important molecular information regarding mechanisms that regulate the acquisition and maintenance of mechanical integrity of the skeleton.

PIN1 is expressed in a wide range of tissues. The prominent expression shown here (Fig. 1A) suggests that PIN1 may play a role in the differentiation and function of osteoblasts and osteoclasts. Analyses of bone marrow cells and osteoclast markers in serum showed the numbers and function of differentiated osteoclasts in *Pin1* KO mice are equivalent to those in WT littermates. However, the number of osteoblasts differentiated from bone marrow were slightly lower (10–15%, data not shown) and the differentiation of primary calvarial osteoblasts were significantly impaired by *Pin1* knock-out. Moreover, the BMP2-induced expression of osteoblast markers (Cbfa1, OSX, ALP, OCN, and COL-I) as well as the formation of bone nodules in vitro were significantly decreased in the absence of *Pin1*. However, there were no detectable changes in the baseline expression of osteoblast markers in whole humerus bone of young mice. This may result from RNA contamination from non-target cell population (e.g. chondrocyte, bone marrow, pre-osteoblasts, fibroblast, and immune cells) that also express osteoblast-related genes but are not regulated by PIN1. Other possibilities may include the various aged mice used experimentally (2–3 week-old for bone marrow study and bone immunoblot, 4 month-old for serum biochemistry, µCT and histology). However, in aggregate, our data strongly suggest that the defect in bone formation observed in KO mice results from defective osteoblast differentiation and function rather than osteoclastic bone resorption.

Mice lacking type I or type II BMP receptor are embryonic lethal [18], [19], indicating BMPs are necessary for normal development. PIN1 KO mice survive at birth but exhibited reduced bone radiodensity in an age-dependent fashion (Fig. 1), and significant loss of trabecular bone in both long bone and spine (Fig. 2B and 2C). Thus, PIN1 is unlikely to be the sole determinant of BMP receptor-mediated signaling. Consistent with this, BMP receptor binding proteins, SMAD inhibitors and activators, and ubiquitin ligases [4] have all been implicated in BMP/SMAD signaling. These factors likely coordinately regulate the overall pathway as seen in many other systems. This is illustrated by our results showing 40–60% reduction of transcription factors and ECM in PIN1 KO mice (Fig. 5) and 6–10% loss of BMD (Fig. 1) both in vitro and in vivo. On the other hand, PIN1 has been implicated in a variety of cascades including NF-kB, WNT, MAPK and hormone-mediated signaling as well as TGF/SMAD pathways [5], [20], [21], demonstrating overlapping functions in different tissues.

Inorganic Pi and Ca^2+^ are essential for skeletal mineralization and a variety of cellular processes. Their serum concentrations are largely determined by kidneys, intestine, bone and hormones. Four month-old *Pin1* KO mice exhibit hyperphosphatemia that normalized in aged mice (18 month). These may reflect age-dependent alterations in intestinal and renal Pi absorption or dysfunction of endocrine systems. Indeed, both PTH [9] and Vt.D3 (Fig. 3D) were significantly overexpressed in the sera of *Pin1* KO mice. Continuously high PTH level is known to trigger bone resorption indirectly through osteoblast activation of osteoclasts via RANKL. However, we have not observed aberrant osteoclast numbers and function in *Pin1* KO mice. This may account for osteoblast dysfunction in PTH receptor signaling in the production of RANKL. The effects of Vt.D3 on bone (anabolic and catabolic) depend on its dosage and the conditions (e.g. severity of deficiency, serum calcium level and bone turnover state) under which it is administered. Mice lacking Pin1 are physically healthy with normal serum calcium and at 4-month the mice are not in active bone remodeling. Thus, the effects of Vt.D3 at higher concentrations seen here could maintain or even stimulate bone formation. Despite elevated Pi (Fig. 3B), Vt.D3 and PTH levels, the mice exhibited normal Ca^2+^ levels, decreased ALP activity (Fig. 3), and reduced BMD and bone volume, suggesting the biochemical changes may be attributable to osteoblast dysfunction that might be the major factor contributing to in vivo bone loss rather than endocrine factors or osteoclastic bone resorption. Our data also indicate that PIN1 plays an essential role in normal and pathologic Pi, Ca^2+^ and endocrine homeostasis.

Various transcription factors and signaling molecules have been identified as substrates for PIN1 [5]. For example, PIN1 enhances AP-1 activity via isomerization of both c-JUN and c-FOS. We demonstrated that PIN1 isomerization modulates the pro-apoptotic activity of BAX and the mRNA binding ability of AUF1 thereby influencing immune cell death and cytokine production associated with allergic asthma [6], [7], [8]. The BMP-regulated SMADs have several potential PIN1 sites in the linker domain, a region which bridges N-terminal MH1 and C-terminal MH2 domains. We propose that osteogenic factors induce linker phosphorylation in premature osteoblasts enhancing PIN1 interactions. As PIN1 expression and its activity were increased after BMP stimulation, this may drive a conformational change in SMAD5, maintaining its phosphorylation, hetero-oligomerization with SMAD4, localization or transactivation potential in osteoblasts. Indeed, recent studies revealed TGF-β induced metastasis was regulated by/through PIN1 interaction with the SMAD2/3 linker [20], [21]. As TGF-β is a potent inhibitor of terminal osteoblast differentiation and abundantly deposits in bone matrix [22], [23], it would be intriguing to pursue the role of PIN1 in TGF signaling in bone. Taken together, our findings in present study suggest that PIN1 is crucially important for signaling mediated by TGF-β super family and could be a potential drug target for the treatment of low/high bone mass diseases.

## Supporting Information

Table S1 **Relative expression levels of osteoblast marker genes in MC3T3E1 cells.**
(TIF)

Table S2 **Mouse qPCR primers used in present study.**
(TIF)

Figure S1 ***µCT morphometric analysis* of trabecular *bone in distal femurs.*** (***A***) Masson’s trichrome staining of mouse femurs to visualize collagen (blue) and trabecular bone. (B-E) After scanning bone with CT, trabecular parameters were determined by MicroView 3D Image Viewer. *denotes p<0.05 by paired student t-test with 6 mice (2 females and 4 males) per group.
(TIF)
