# Supplementary figures and images for: Pin1 Null Mice Exhibit Low Bone Mass and Attenuation of BMP Signaling
Source: PLoS One. 2013 May 10;8(5):e63565. doi: 10.1371/journal.pone.0063565 (PMC3651169; doi:10.1371/journal.pone.0063565)

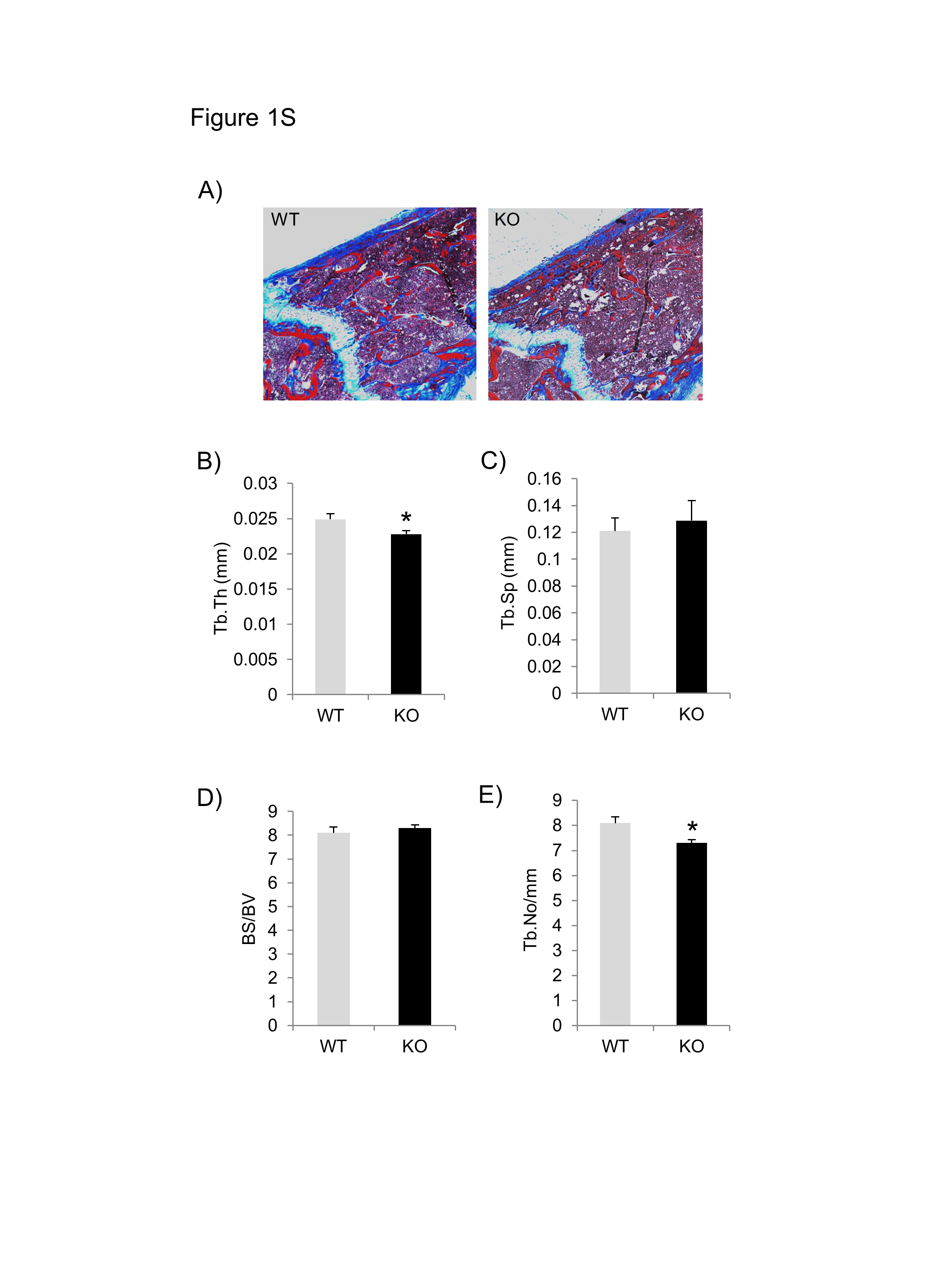

Supplement: Figure S1 — µCT morphometric analysis of trabecular bone in distal femurs. (A) Masson’s trichrome staining of mouse femurs to visualize collagen (blue) and trabecular bone. (B-E) After scanning bone with CT, trabecular parameters were determined by MicroView 3D Image Viewer. *denotes p<0.05 by paired student t-test with 6 mice (2 females and 4 males) per group. (TIF) [file pone.0063565.s003.tif]
